# Legume dreams: The contested futures of sustainable plant-based food systems in Europe

**DOI:** 10.1016/j.gloenvcha.2021.102321

**Published:** 2021-07

**Authors:** George Cusworth, Tara Garnett, Jamie Lorimer

**Affiliations:** a Oxford Martin Programme on the Future of Food, c/o Oxford Martin School, University of Oxford, 34 Broad Street, Oxford OX1 3BD, UK; b Hertford College, Cattle Street, Oxford OX1 3BW, UK

**Keywords:** Legumes, Food systems, Futures, Promissory narrative, Livestock, Europe

## Abstract

With the intensification of agriculture, the simplification of crop rotations, and the rise in demand for meat, dairy and cereal products, legume production and consumption are at an historic low in Europe. But as the environmental consequences of agriculture (biodiversity loss, high greenhouse gas emissions, water pollution) and the health outcomes of modern diets (heart disease, cancer, diabetes, obesity) become better known, so great and varied hopes are being expressed about the future role of legumes in the food system. This paper catalogues and scrutinises these hopes, mapping the promissory narratives now orbiting around legumes. It identifies six food futures, each of which is made possible through the greater use of legumes in various production, processing, marketing and consumption contexts. These promissory narratives are theorised as contrasting responses to three major areas of contestation in the food systems literature. Namely i) the sustainability of livestock management, ii) the role of technology in different visions of the ‘good diet’, and iii) the merits of different models for how to make agricultural management more sustainable. It identifies the promiscuity of legumes – in terms of the range of food futures they permit – before distilling three points of consensus amongst advocates of the potential of legumes. These points of consensus relate to their nitrogen fixing capacity, their high protein content, and their long-standing historical role in the context of European food and farming. This map of legume dreams serves to guide deliberations amongst researchers, policymakers and industry stakeholders about the futures of plant-based food in Europe.

## Highlights

• The paper maps the different promissory narratives associated with legumes.
• Legumes are important for both sustainable intensification and agroecological models.
• They can displace meat in our diets and reduce the GHG emissions of livestock systems.
• They can be eaten whole and unprocessed or as processed meat-analogue products.
• Legumes’ versatility enables many different visions of the future of food.

## 1 Introduction

Legumes and pulses are eaten and used for animal feed in many parts of the world. Notable examples include alfalfa, clover, beans, peas, chickpeas, lentils, soy and peanuts. These have long been a part of Western diets and agricultural management regimes, but they have very recently risen in popularity in agri-food research. Promoted for their agronomic, nutritional and environmental benefits (Voisin et al., 2014, Oliveira et al., 2019), legumes have been framed as plant-based solutions to an array of problems in the modern food system; becoming vegetable vessels that express the hopes and dreams of diverse researchers, marketeers and other food futurologists.

For example, agronomic research has explained how legumes can increase soil organic matter and add resilience to crop rotations (Ebert, 2014; Wu et al., 2017; Considine et al., 2017), while increasing crop yields and/or profitability (Luce et al., 2015, Preissel et al., 2015, Reckling et al., 2016). For nutritionists, legumes represent healthy, high-protein and nutrient dense foodstuffs (Tharanathan and Mahadevamma, 2003) that can reduce the risk of heart disease and stroke (Margier et al., 2018) especially as meat-alternatives (Becerra-Tomás et al., 2013). While environmental research shows how the nitrogen fixing qualities of legumes can lessen the greenhouse gas emissions of arable production by lowering the need for mineral fertilisers (Lampkin et al., 2015), while decreasing local environmental impacts associated with diffuse water pollution and runoff (Lotjonen and Ollikainen 2017*)*. When eaten in place of meat and dairy, legumes have the potential to reduce pressure on agricultural land and resources (Day, 2013, Röös et al., 2017) and to address the high GHG emissions associated with livestock management (Davis et al., 2010, Jones and Ejeta, 2016).

In spite of these numerous and varied promises, the production and the consumption of legumes have been in steady decline since the second half of the twentieth century (Hedley, 2001, Zander et al., 2016). This mismatch between current reality and anticipated potential has catalysed a wide range of research, development and marketing activity. In Europe, for example, several well-funded multinational projects (with titles like LegumeValue, LegGap, ReMIX, and LegumesTranslated) have been established to examine the agronomic applications of legumes, to close yield gaps, and to assess how they might be able to improve the environmental profile of European farming. This uptick in research is matched by early signals of growing consumer appetite for legumes (or processed products that used legume-derived ingredients) driven by the rise of plant-based and flexitarian diets (Dagevos, 2014, Metzger et al., 2013).

The main aim of this paper is to make sense of the varied aspirations now orbiting around legumes. We do this by providing a map of their ‘promissory narratives’, as broadcast through different academic, media and food marketing communications. As the voices promoting the use of legumes in the creation of a healthy and sustainable food system intensify, and as their claims diversify, the paper is designed to help cut through the noise and make explicit the political and cultural sensibilities of legumes’ advocates, and the different food futures they are called on to legitimise.

## 2 Promissory narratives and food futures

Following Sexton et al. (2019), we understand the concept of a promissory narrative to refer to a story of a future that will be made possible by a technology, usually as a remedy to an environmental or social ill. The term is predicated on sociological work that grapples with the ways in which potential solutions to specific problems are brought to life through imagined future scenarios – imaginations typically deployed for commercial or political gain (Brown and Michael, 2003). This understanding builds on Anderson’s (2010) work on the ways in which representations and predictions of the future legitimate anticipatory action. The future is, in this way, brought into active and normative dialogue with the present; articulating which courses of action will lead to which outcomes, foreclosing on alternatives, and thus prescribing how to act now to avoid a prefigured eventuality. The paper deploys this conceptual framework to develop the idea of ‘food futures’ as they are described in more policy-orientated studies (Ambler-Edwards et al., 2009). We define a food future as an imagined arrangement of the key forces in food and farming – including consumers, farmers, politicians, researchers, and supermarkets – that is presented as a more or less feasible aspiration for what the food system could look like. Whilst promissory narratives and future imaginaries are often attached to technological fixes (Jasanoff, 2015, Goldstein, 2018), in the context of food and farming, the language of salvation is deployed across a diverse range of low-tech and high-tech, scientific and spiritual, traditional and modern practices (Sexton et al., 2019, Kawa, 2020, Kearnes and Rickards, 2020).

The need to provide this promissory roadmap arises because legumes have largely been denied critical attention. Whilst there is much research looking into their health and agronomic advantages, and whilst many actors are now seizing on these qualities to animate different promissory narratives, how legumes fit with broader critical thinking around sustainable food systems remains unclear. They are, put differently, well researched but under-theorised. This situation is manifest in the diversity of promissory narratives we present in this paper. Legumes – and their greater representation in the food system - are being used to legitimise a broad range of contradictory, even antagonistic food futures. They thus emerge as a promiscuous plant, permitting many different perspectives on how food production, marketing, processing and consumption should be orchestrated.

This lack of theorisation matters. Elsewhere in the literature, social science research has demonstrated how promissory narratives have the power to anticipate and shape the trajectories of agricultural innovation and land use change (Goodman, 2004, Evans and Miele, 2012, Sexton et al., 2019). In presenting a map of these narratives – or what we term legume dreams – the paper is looking to extend this work: to refine our thinking around legumes, what role they are being asked to fill in different food futures, and by whom. In conclusion we develop this critical thinking further. We reflect on the points of consensus between different promissory narratives and explore how these might form the basis for deliberation on the priorities for future research, policy and innovation. The paper is primarily European (UK included) in scope, but the arguments presented here are relevant to other regions where legumes are being promoted or that will be impacted by the rise of plant-based diets.

## 3 Methods

To undertake this mapping work, we reviewed academic journal articles, research projects, policy documents, media, and food marketing outputs. We did not conduct a systematic review, in which all relevant materials are identified and compiled, but a qualitative, narrative review. Following the methods described in Kitson et al. (2012), we were able to identify and juxtapose different promissory narratives in circulation, going beyond a purely descriptive distillation of the available literature.

The review was completed over the spring and summer of 2020. Initially, conventional academic literature search tools (Google Scholar and Web of Knowledge) were used, guided by permutations of the search terms: legumes, pulses, processing, agronomy, crop rotations, nitrogen fixation, farming, health, sustainable diets. The references in the papers returned in this search were also followed up and, where relevant, were included in the review. The coding process of these documents was initiated by the themes ‘promises’ and ‘futures’ to deliver on the paper’s central conceptual design. Doing so helped reveal how various food futures were implied or made explicit across different research fields. Following Lavallée et al.’s (2013) description of iterative literature review techniques, a series of rounds of coding saw the emergence of different promissory narratives clustered around constellations of health, agronomic, and food processing claims. This process was continued until a saturation point was reached at which a coherent set of six promissory narratives had crystallised.

To ensure that these food futures existed beyond the confines of academic exchange, these narratives were then overlaid and populated with the findings of a more purposive review of the media and marketing coverage of legumes. This aspect of the review helped reveal the epistemic community behind each promissory narrative: disclosing who was making what claims, with what authority, and what cultural sensibilities were at work in their promotion. For this component of the review, we employed Lupton’s (2017) methodology for using the Google News search tool in academic research. Our review covered mainstream media outlets, as well as dedicated food and farming periodicals.

The findings of this review were then used as a departure point for interviews with a sample of 10 relevant stakeholders in the UK food sector. Interviewees were identified through a purposive methodology. They were selected for their professional proximity to legume production, marketing and consumption; or because they could speak to their status as objects of agronomic, health and food processing research. Interviewees were able to corroborate the conclusions of the review and to refine our map of promissory narratives. These interviews thus functioned as a member’s check (Birt et al., 2016), improving the robustness of the analysis.

## 4 Legumes in the European food system

This section provides an introduction to legumes and their status in the European food system. This is a context-setting exercise, separate from the main, promissory narratives, component of the paper. Although the narratives we discuss look to rewrite, reimagine or revitalise their past differently, this history cuts across their respective boundaries.

Legumes are the edible seeds from the Leguminosae family. The term also serves as the general name for the plants themselves. There is some confusion about the difference between legumes and pulses (Vollmann, 2016). Pulses are a subset of the legume family. Their seeds are dried before being eaten. Examples include chickpeas and lentils. Examples of legumes that are not pulses include fresh pea, soy and peanut, as they are harvested in their green state and are eaten whole or processed for their flour, oil or meal. Other legumes can be grown for forage or used for sileage. These include lucerne (alfalfa), different clovers and bird’s-foot trefoil.

**Fig. 1:**
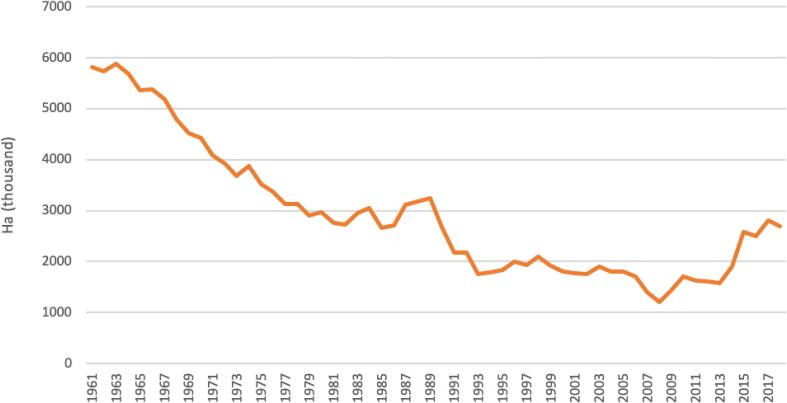
European legume cropping area over time, data taken from Eurostat (2020).

Legumes have provided cheap and high-protein food throughout European history (Flint-Hamilton, 1999, La Poutré, 2015), especially in Mediterranean cultures (FAO, 2016). They were once one of the major crop types in Europe, playing an important role in the diets of peasants and serfs and helping to catalyse advances in agricultural output by fixing nitrogen (Stinner et al., 1992, Allen, 2008). Mixed farming systems with longer and more varied rotations that included legumes were common (Neuenfeldt et al., 2019, Zander et al., 2016). But since the 1950s, a number of factors have driven a decline in European legume production. Key shifts include the high prices available for cereals (prompting farmers to commit more land to their production); the availability of cheap imported animal feed (reducing the need for local supply chains of feed crops and forage); developments in agricultural machinery (larger machinery designed for cereals cultivation and not legume cropping); a policy landscape that rewarded high-yielding crops (price guarantees); and the availability of mineral nitrogen inputs (reducing the need for legumes’ nitrogen fixation) – processes described in detail by Zander et al., 2016, Magrini et al., 2016, and Watson et al. (2017). Fig. 1 shows the total cropping area of legumes in Europe (UK included) over time. Note the continual decline over the second half of the 20th century and the uptick in the early 2010s that was largely catalysed by the introduction of crop diversification rules with the EU Common Agricultural Policy.

**Fig. 2:**
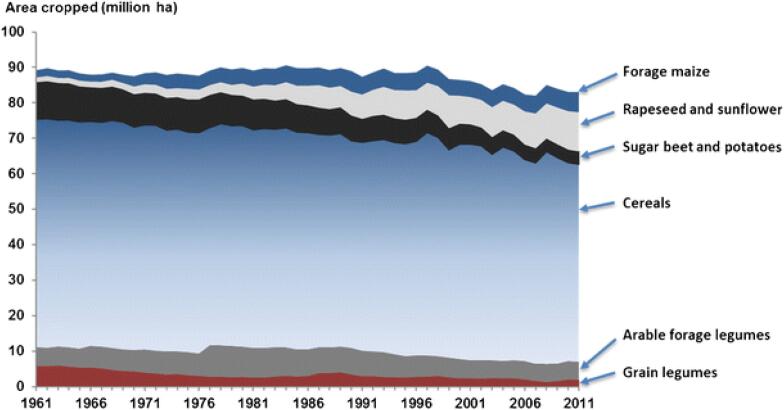
The relative cropping areas of different agricultural products in the EU (data taken from Zander et al. 2016).

Historians explain how the structural changes listed above are part of the general intensification and specialisation of agriculture, the loss of mixed farms, and a simplification of crop rotations (Stinner et al., 1992, Robinson and Sutherland, 2002, Rochon et al., 2004, Meynard et al., 2018). Legumes now account for only 1.5% of the cropped area of Europe down from 4.7% in 1961 (Mahmood et al., 2018) (Fig. 2). In Europe, legume production is only one tenth of the global average for legume production on arable land.

**Fig. 3:**
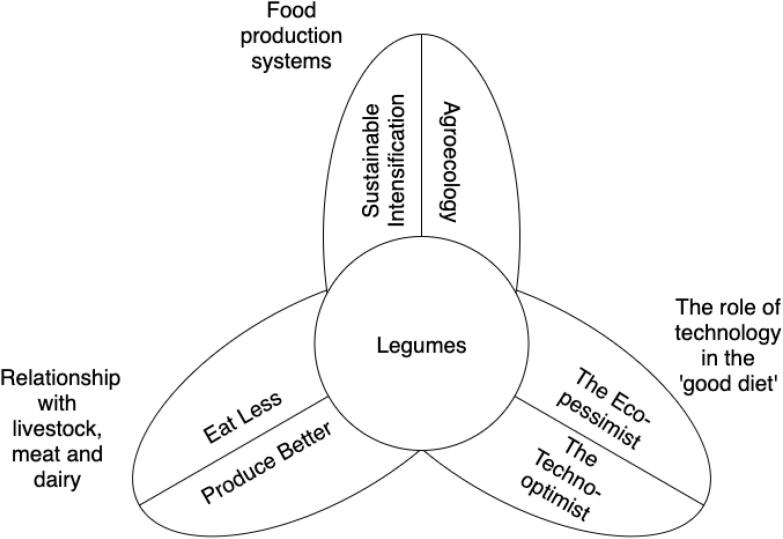
A schema showing six promissory narrative of legumes mapped onto 3 areas of contestation.

Linked trends can be observed in the demand for legumes, which has also been in steady decline since the second half of the 20th century (Hedley, 2001, Oliveira et al., 2019, de Souza Monteiro et al., 2017) (Fig. 3). De Boer *et al.,* calculate that consumption of protein from legumes in the EU-15 Member States ranges from 0.1 g to 3.7 g per person per day, out of a total protein consumption of between 96 g and 119 g (de Boer et al., 2006). The general post-WW2 rise in wealth and disposable income, coupled with their increasing cheapness and availability, led to the concentration of calorie and protein consumption in Europe in cereals, meat and dairy (Gerbens-Leenes and Nonhebel, 2002). This shift was amplified by the aspirational attraction and status of meat (Macdiarmid et al., 2016)*,* and the stigma of legumes as ‘poor man’s meat’ (Tharanathan and Mahadevamma, 2003).

This decline is tied up with their low productivity. The focus in the last half-decade on improving chemical inputs, cultivars and machinery has led to considerable improvements in the yields of cereals and oilseeds but stasis in legume yields (Magrini et al., 2016, Metzger et al., 2013, Squire et al., 2019). This research marginalisation is manifest in the lack of climatically suited high-yielding cultivars, developed supply chains, agronomic knowledge and targeted agricultural products (pesticides, machinery) to assist production (Magrini et al., 2016, Zander et al., 2016). As European farm practices and their business models became dependent on high-yielding and intensive systems for cereals, oilseed, specialist dairy, pork, beef production, farmers became ‘locked-out’ of using lower-yielding legumes, making production financially untenable and socially undesirable (Magrini et al., 2016, Magrini et al., 2018, Meynard et al., 2018).

There is a sense amongst enthusiasts that this marginal position is shifting. In the introduction to a special issue of *Experimental Botany* dedicated to legume research, Considine et al. (2017) present legumes as enjoying a ‘coming of age’ moment; a maturity evident in a vast array of legume productivity research and their food processing potentials. Broad interest in crop rotation sustainability, drought resilience, flood risk mitigation, and resilience to pests and disease are animating this legume research agenda. The development and research processes that have improved wheat, rye grass, and oilseed rape yields are, in other words, being applied to legumes with the express purpose of making them a resilient and reliable tool for delivering on new food security and climate change challenges, to close yield gaps, and to recover ground they have lost to other cash crops in the last seven decades of agricultural research.

Similar enthusiasms are expressed on the demand side. Recent market forecasts have documented a modest increase in legume consumption which is predicted to grow further due to the demand in health food markets (Redman, 2015) and the mainstreaming of vegetarian and vegan diets (Morris, 2018, Clay et al., 2020, Mann and Necula, 2020)*.* A Research and Markets report forecast a 4.6% growth in the global pulse market from 2019 to 2027 (Research and Markets, 2020) – motivated by markets for whole foods, meat-alternative products, bio-fortification with legume grains, and ready-to-eat meals targeted at health and environmentally conscious consumers (Redman, 2015, Jha and Warkentin, 2020). As market incentives from food labelling to agricultural subsidies are increasingly predicated on delivering food with higher environmental standards, so a broad range of producers and processors have been given reason to see how legumes can fit within their product portfolios.

## 5 Legumes and their promissory narratives

Promissory narratives are, in part, brought to life by the problem they are trying to solve (Brown and Michael, 2003, Sexton et al., 2019). The narratives in our review emerged in relation to three of the major tensions present in current discussions around the operations of the contemporary food system. We use these issues to organise and present the findings of our review. They are i) the sustainability of meat and dairy; ii) the role of technology in food processing and marketing; and iii) the merits of different models of agricultural management. In each instance, legumes feature heavily in two contrasting ‘solutions’ to the problem (Fig. 3). They are thus being enrolled into a broad – and conflicting - range of food futures. In the first tension, we examine debates over the environmental damage caused by livestock and the consumption of meat and dairy and compare solutions geared towards either reducing consumption or producing at a higher environmental standard. In the second, we look at the role afforded technology in food processing and marketing, comparing an eco-pessimist with a techno-optimist perspective on the ‘good diet’. In the third, we examine debates over how to make farming more efficient, productive and less environmentally damaging, comparing two of the most prominent solutions: Sustainable Intensification and Agroecology

We present these six positions as brief, somewhat caricatured, strawman depictions. This gives the paper room to cover significant ground in the literature and to foreground areas of political and epistemic disagreement. It is important to note that describing these six positions in such a mutually exclusive and contrarian way is a heuristic device. In reality, each position has several different and more nuanced incarnations. In the paper’s conclusions, we look to go beyond these simplified depictions by discussing the areas of consensus, even between apparently contradictory food futures. This ‘reading for difference’ (Gibson-Graham, 2006) offers a way of initiating investigation into agreement and overlap – to disrupt the binary, zero-sum tone that sometimes threatens to take hold in exchanges around ‘good’ food and farming.

### 5.1 The place of meat and dairy: Reducing consumption or greening the livestock supply chain

The production and consumption of meat and dairy are one of the most febrile points of discussion in the food systems literature today. Livestock farming contributes 14.5% of global anthropogenic GHG emissions (Gerber et al., 2013). Within Europe, the livestock sector contributes 12–17% of the territory’s total emissions (Bellarby et al., 2013) and is responsible for 85% of agricultural emissions (Leip et al., 2010)*.* These emissions come in the form of enteric methane production, primarily from ruminant grazers but also from swine and poultry systems (Ellis et al., 2010, Wang and Huang, 2005, Daemmgen et al., 2012). The use of mineral fertiliser to promote the growth of high-yielding grasses also contributes nitrogen to the atmosphere, and CO2 from the energy used in production (Bentrup and Palliere, 2008). Through the demand for feed and grazing land, livestock farming also drives land use change and the release of stored carbon (Fraanje and Garnett, 2020). The production of livestock is also understood to be inefficient in terms of both water and land use (Gadanakis et al., 2015, Springmann et al., 2018). Livestock agriculture’s environmental footprint can also be understood as an opportunity cost: the land used for feed and grazing could be used for rewilding, afforestation or other carbon sequestration practices (Garnett, 2009, Herrero et al., 2011)*.*

As concerns about climate change have become mainstream, and as these contributions of agriculture have become better understood, so the pressure to reimagine the role of livestock systems has grown (Budgar, 2017). Steinfeld and Gerber (2010) identify two broad sets of policy responses to the problem of livestock emissions: to consume less or to produce better*.* The first aims to reduce the share of meat and dairy in diets and thus the land dedicated to their production. The second aims to fix the environmental problems embedded in the supply chain, especially those associated with intensive livestock farming.

#### 5.1.1 Produce better

Advocates of the first approach have focused their attentions on animal feed (Watson et al., 2017, Rochon et al., 2004), which is one of the main causes of GHG emissions in the European livestock sector (Weiss and Leip, 2012). Unless ruminant animals are raised exclusively through extensive grazing on either temporary or permanent grass, their diets are supplemented with feed. There has been a structural shift in farm management from a system characterised by feed self-sufficiency and the utilisation of on-farm by-products to an intensive and specialist model predicated on products brought onto farms along international feed and fertiliser supply chains (Gruber and Galloway, 2008, Galloway et al., 2008).

In feed terms, Europe is only 30% self-sufficient and 87% of that deficit is made up by imported soy and soybean meal (Watson et al., 2017). Europe currently imports 20 million tons of soybean meal and 12 million tons of soy grain annually, primarily for poultry and pork, but also ruminant systems (Mahmood et al., 2018). This lack of protein self-sufficiency in the livestock sector is an important cause of Europe’s ‘ecological hoofprint’ (Weis, 2013; Weiss and Leip, 2012). Most of the soy imported for feed comes from South America (Weightman et al., 2011) where demand for feed has been a key driver in land use changes that have led to deforestation and the release of vast amounts of carbon stored in the soil and trees (Oliveira and Hecht, 2016). Although the task of diagnosing which products are driving what land use change is made more complex by the conversion of forest land to cattle ranching before being used for intensive soy production, demand for soy has affected large areas in the Amazon and the Cerrado (Fraanje and Garnett, 2020). It has been estimated that the land use changes that enable the production of the feed imported to Europe account for around one third of the sector’s total GHG emissions (Bellarby et al., 2013, Weiss and Leip, 2012).

Proponents suggest that improving the protein self-sufficiency of the European livestock system offers an important lever for reducing reliance on imported feed products and reducing livestock GHG emissions (Lesschen et al., 2011, Watson et al., 2017). One way of achieving this is through the use of grass-legume mixes on either temporary or permanent pastures. These mixes involve the establishment of a biodiverse grazing mix of legume varieties such as clovers, lucerne, birdsfoot trefoil and sainfoin and grasses, typically rye (Lüscher et al., 2014*)*. The different plants in the sward have different growth patterns, foliage height, root depth and mineral needs. Relative to the pure grass mixes now common across Europe, such heterogeneity can increase the protein content of the available grazing, reduce GHG emissions by reducing fertilisation levels, and provide excellent nutrition for browsing livestock throughout the year (Lesschen et al., 2011, Ledgard et al., 2009). The farming press is generous and positive about the merits of making such changes in farm management (FWI, 2020), not least because they are seen as a necessary step to demonstrate the sector’s willingness to take the issue of GHG emissions seriously (Farmers Guardian, 2020).

Those promoting legumes as a means to improve livestock systems suggest that the local production of legumes for feed products can also help reduce the environmental footprint of livestock beyond pasture systems. They suggest that compound and processed feed can be produced with European products to displace the current reliance on imported feed products (Watson et al., 2017). For example, a Swedish Life Cycle Assessment replacing imported soymeal with European-grown pea and rapeseed oil-based feed found the potential for a 15% reduction in GHG emissions per unit of milk produced (Sasu-Boakye et al., 2014). A further study of the GHG profile of dairy production in Austria, calculated that using locally produced high-protein legume-based feed reduced GHG emissions by 42% relative to imported soy products of the same nutritional value (Hortenhuber et al., 2011). This promissory narrative makes explicit reference to the long-standing usage of legumes in forage and feed provisioning as a retrospective food future (ORC, 2018).

Those in favour of localising animal feed production in Europe suggest that soy is the most promising legume and point to the successes of recent projects in a number of European Member States. For example, Ionel (2017) report on how the Romanian agricultural sector has established itself as a major European producer of soy by offering additional subsidies for the production of high protein crops. This scheme increased total soy production by 30% (to 260 thousand tons) in 2015. The extent to which locally produced feeds can offset the emissions associated with imported soy-based products depends on the system involved, and the levels of feed bought in. Extensive ruminant grazing, which uses relatively small amounts of feed will be able to make smaller GHG savings than intensive indoor poultry or swine systems (Gerber et al., 2013, Leip et al., 2010).

#### 5.1.2 Eat less

In contrast, a second set of advocates have explored the potential of legumes to reduce the consumption of meat and dairy. Legumes are frequently identified as nutritious dietary substitutes (or ‘meat alternatives’) central to the curation of sustainable diets. These messages find voice across social media (Pohjolainen and Jokinen, 2020), food marketing (Hamann et al., 2019) and academic communications (Davis et al., 2010, Röös et al., 2018, Springmann et al., 2018). The protein content of legumes sits between 17 and 30% of their dried weight (Boye et al., 2010) – much higher than most other plant foodstuffs – meaning they are ideal products for inclusion in a diet not orientated around meat or dairy (Rebello et al., 2014). In carbon terms, the production, packaging and distribution of a kilogram of pulses amounts to 2% of that used to produce a kilogram of lamb, 3% of that used for a kilogram of beef, and 16% of that used for a kilogram of pork (Global Food Security, 2017*).* Proponents of using legumes as meat alternatives argue that the environmental imperative to reduce meat and dairy consumption is especially acute in Europe (and the rest of the Global North) due to the higher than global average consumption (Boland et al., 2013, Vranken et al., 2014).

Other advocates argue that an increase in the consumption of legumes in place of meat in European diets will have health benefits (Curran, 2012, Margier et al., 2018). Many legume crops are high in dietary fibre (Trinidad et al., 2010), low in fat (Rizkalla et al., 2002, Asif et al., 2013), are rich in a range of micronutrients (Mudryj et al., 2014) and have been linked to a lower risk of cardio-vascular disease and stroke (Anderson and Major, 2002, Curran, 2012). The nutritional, fibre and protein content of legumes give them a high satiety index (making the eater feel full) (McCrory et al., 2010), and so they provide useful tools for dietary self-regulation and weight management. Legumes are particularly important for vegetarian and vegan diets owing to their relatively high lysine content. Lysine is an amino acid essential for muscle development, metabolising fat, calcium absorption and producing collagen (Pellett and Ghosh, 2004) that many people obtain through meat and dairy consumption. Legumes, especially chickpeas, soy, lentils and beans, are some of a limited number of foodstuffs capable of delivering requisite levels of lysine in meat reducing diets (Iqbal et al., 2006, Röös et al., 2018). These messages occupy central roles in the marketing materials of the rapidly growing list of plant-based foods using legumes-based ingredient lists (Euvepro, 2019).

In this section, we have documented how legumes have returned to prominence in food systems research as increasing attention is paid to the environmental effects of livestock and the need to reconcile global dietary trends with human health outcomes and production sustainability (Joshi and Rao, 2017). Taken together, the research and advocacy reviewed above suggests that legumes can help address these dual pressures exerted on human and environmental health (Foyer et al., 2016). In the language of Steinfeld and Gerber (2010), they can help us both eat less and produce better. For advocates, their environmental benefits – both in terms of their capacity to facilitate a reduction in meat and dairy consumption, and their capacity to mitigate the environmental externalities of livestock management – give good reason to increase their representation in the food system (Nemecek et al., 2008, McDermott and Wyatt, 2017).

### 5.2 Legumes and technology: eco-pessimist and techno-optimist visions

As the pressure grows to reconcile dietary patterns with agricultural sustainability, so individual meat and dairy consumption have become subject to greater public discussion (Beverland, 2014, Rosenfeld and Burrow, 2017). Two distinct narratives are emerging from these debates – that we might describe as the eco-pessimist and the techno-optimist – both of which find good reasons to look to legumes to help reconcile dietary choices with environmental objectives. Eco-pessimism and techno-optimism are terms that denote broadly differing understandings of human relationships with nature, the causes and solutions to today’s environmental crises, and most importantly the desired role of biotechnology.

#### 5.2.1 Eco-pessimism

Eco-pessimism is founded on the idea that modern society is disconnected from nature. It is a political philosophy with a long cultural history, linking late eighteenth-century Romanticism with twentieth-century Deep Ecology (Worster, 1994, Radkau, 2014). For eco-pessimists this alienation is the result of conflating the idea of human progress with technological advances and the growth of consumption (Parham, 2015)*.* Certain technologies, in this framing, are an inherent feature, perhaps even a cause of the broken relationship with the natural world that underpins today’s environmental problems. Technological trends in the food system that are taken as symptomatic of this disconnection include the intensification of agricultural production, the increased reliance on agricultural chemicals, genetic modification, the loss of synergistic crop rotations, the rise of international agricultural commodity markets and the loss of local and seasonal specificity. This political and aesthetic positioning can be seen across a range of media (The Guardian, 2019), advocacy (Landworkers’ Alliance, 2014) and literary (Rebanks, 2020) channels.

Critics suggest that the fact that the environmental damage caused by these developments were unforeseen at the time of their development should caution against any temptation to go further down the rabbit-hole of agricultural modernisation, specialisation and intensification (Basu and Scholten, 2012, Friends of the Earth, 2012). In other words, hubristic humans should not look to further innovate their way out of current problems by creating new high-tech agricultural products and food processing systems, by increasing reliance on GM crops, or by further extending international global supply chains. Instead the aim should be to simplify food production and consumption systems returning to more ‘natural’ forms that emulate traditional or historical practices.

Legumes have been enrolled in such eco-pessimist visions. In food marketing, legumes are framed as a wholesome, clean, environmentally friendly and humble product (Hexa, 2019). They are stocked in health foods and wholefood stores across Europe and the Global North (de Souza Monteiro et al., 2017, Redman, 2015), that often position themselves as the traditional, pure, local and just alternatives to supermarket chains (Lockie, 2009, Seyfang, 2006). In caricature, legumes are emblematic of the ascetic food choices that vegetarians and vegans had (until recently) to make to fulfil their moral dietary choices. Lentils, chickpeas and beans represent the sacrifices you have to make to make your diet more sustainable – the proverbial food of the hair shirt wearing eco-fundamentalist (Smart, 2004)*.*

Eco-pessimists promoting legumes in the European diet to fix agriculture’s environmental and social problems present them as vehicles for the revitalisation of traditional, low intensity production systems, for the reinvigoration of diets based on unprocessed foods, and for enabling a return to distribution networks based on direct and short supply chains. They make reference to the historical role of legumes as a baseline to justify future expansion.

For example, Hodmedods (a UK-based legumes producer) champion their fava beans and peas as a return to a traditional and historical version of the British food system. In a recent blog post, one of the founders leans on this historicity to promote fava beans: ‘Introduced to Britain by the first Bronze Age farmers, they were among the earliest farmed crops alongside wheat, barley, oats and peas. Harvested dry and readily stored, the beans provided an excellent and reliable year-round source of protein’ (Pulses UK, 2020). Similar retrospective futures characterise the promotional narratives told in others parts of Europe (Kulak et al., 2015), with legumes featuring in alternative food networks in Italy (Tudisca et al., 2014, Orlando, 2018), France (Dubuisson-Quellier and Lamine 2008) and Portugal (Harper and Alfonso, 2019).

#### 5.2.2 Techno-optimism

The techno-optimist position is, by contrast, one that stresses the importance of using all modern scientific knowledge and technology available to improve food and agriculture. Science and technology do not disconnect humans from nature but confer the power to control and optimise ecological systems. In the world view of ecomodernism, science and technology enable humans to assume their Enlightenment destiny as the ‘God Species’ (Lynas, 2011) and to solve the environmental problems inherent to modern agriculture. Vertical farming, agricultural robots and the growth of muscle tissue for in-vitro meat production are exemplary of this ecomodernism: harnessing and optimising natural processes for a more sustainable programme of food production (Stephens, 2015, Newman, 2020).

The techno-optimist does not arrive at the legume for their purity and simplicity. Legumes are enrolled into this food future for their versatility and fungibility. Legume derived protein is cheap relative to meat, eggs and dairy, and cutting-edge research conducted in new centres for food technology innovation is demonstrating how legume derived proteins are amenable to a wide variety of processing applications. Legumes can produce convincing meat-like textures for new food products (Makri et al., 2005). Pea, soy, chickpeas and beans have become key ingredients in the proliferating market for vegetarian or vegan meat-simulacra products, which are promoted as solutions to the environmental impacts of livestock farming (Joshi and Kumar, 2015). Highly processed plant-based products such as the Impossible Burger and other smaller or supermarket home-brand alternatives develop the high-protein content and food processing potential of legumes to develop cheaper, greener products. The same processes are also being applied to dairy products. Soy, and to a lesser extent, pea products enjoy a significant share of the growing dairy-free, ‘mylk’ market (Gambert, 2019). They are also being explored for their biofortification potential (Jha and Warkentin, 2020). In such instances, the nutritional properties and sustainability credentials of more recognisable food stuffs (breads, cakes, ready-meals) are advanced by the inclusion of legumes in various processed forms (Kumar and Pandey, 2020, Euvepro, 2019).

These products enable consumers to improve the environmental profile of their diets, without having to make onerous decisions to shift to unglamorous, traditional wholefoods. The increased profitability of meat-free and dairy-free products, derived from a growing consumer base, has given food scientists and retailers good reason to commit their efforts into developing increasingly convincing and marketable products, and the availability, choice and cheapness of those products has attracted a new and mainstream audience (Euvepro, 2019). Such products permit a ‘palatable disruption’ (Clay et al., 2020) to the food system: they are developed and marketed to mimic recognisable and familiar meat products (hence ‘palatable’) but by using a plant-based ingredient list, these products offer an environmentally friendly option (hence ‘disruption’). In the techno-optimist ideology that informs this alternative protein industry, the environmental benefits of legume production (relative to livestock) paired with their processing and nutritional potential have made them a natural ‘fix’ for the environmental problems of livestock.

In this section we have traced how legumes are marketed as foodstuffs that can occupy diametrically opposed spaces in the food landscape. They have, in this reading, a dual personality. They can be the staples of the eco-pessimist who would prefer the revitalisation of unprocessed, traditional and whole foods to help minimise the negative environmental externalities of food production. They also serve as the fungible base materials for high-tech food processors who aim to produce convincing meat-simulacra to displace unsustainable meat and dairy products without fundamentally changing culinary practices.

### 5.3 Legumes and intensification: Making food production sustainable

There is a well-documented need to increase the availability of food whilst decreasing the environmental cost of production (Godfray et al., 2010). Different actors have presented legumes as amenable to two of the major responses to the problem: Sustainable Intensification (SI) and agroecology. SI focuses on increasing the output of the global food system (nutrients, calories, protein, food security and accessibility) whilst reducing its inputs (land use, water use, agricultural chemical inputs) and improving its environmental profile (biodiversity loss, GHG emissions, water pollution) (Godfray and Garnett, 2014). Since the concept’s inception in a 1997 study looking into productivity in small-holding farms in Africa (Pretty, 1997), it has gained significant traction as a policy paradigm, and now occupies an important space in most national (EU Member States included) and international (the FAO, the EU) food and farming strategies (Tomlinson, 2013, Friedrich, 2015, Staniszewski, 2018).

There is ongoing debate about which practices enable SI (Mahon et al., 2017, Weltin et al., 2018). But, in theory, anything that helps increase agricultural productivity and yields whilst reducing negative externalities falls under its rubric (Wezel et al., 2015, Pretty et al., 2018). In the recent incarnations of the theory, that will be used for the purposes of this part of the paper, SI involves innovations like integrated pest management, biotechnologies such as high yielding and pest resistant cultivars and animal breeds, and precision farming. An SI food future fosters the development of global supply chains, and the regional specialisation of food production, leveraging the comparative advantage of agricultural production in different parts of the world. In this vision of the future of food, there is an inherent prioritisation of *intensification*: we do not need to critically reimagine how food is distributed, sold or consumed, but rather to fast-track the integration of systems and technologies that can fix the negative environmental impacts of production. Insofar as it permits a largely unchanged focus on scale and productivity (albeit it with improved emissions efficiencies), this model of agricultural development is amenable to farming psychologies (Dicks et al., 2018) and the politics of the major voices in the farming mainstream (FWI, 2014, NFU, 2016).

In contrast, agroecology is the practice of integrating ecological principles into farm management to emulate the self-regulation and homeostasis present in the natural world to reduce the negative environmental externalities of food production (Gliessman, 2007). Advocates for agroecology critically interrogate the modern logics of intensification to avoid repeating the environmental mistakes of the last 70 years of agricultural development (Friends of the Earth, 2012, Bernard and Lux, 2017). As with sustainable intensification, the precise perimeters of agroecology are not well defined (Wezel et al., 2009) – but organic management systems, veganic systems (stockless organic), no-till management, intercropping and cover crops all typically fall under its rubric. In a broader debate about the food system, and in specific contrast to sustainable intensification, agroecology also emphasises the need to: foster small and local supply chains to reverse the current trend of consolidation in food production, processing and distribution; make broader ecological objectives central to agricultural production; redistribute power in the food system; and revitalise a connection with seasonality, locality and the relationship between the producer and consumer (Holt-Giménez and Altieri, 2013, Méndez et al., 2013).

Although the two paradigms of sustainable intensification and agroecology represent divergent visions of how sustainability, globalisation and intensification should be reconciled (Horlings and Marsden, 2011), both place considerable stock in the ability of legumes to advance their objectives. In particular they share a common interest in the capacity of legumes to fix nitrogen from the air into the soil (Gan et al., 2015), and their resulting ability to displace the need for mineral fertilisation inputs (Stagnari et al., 2017) and thus to reduce GHG emissions. However, they have contrasting visions for how this benefit might best be delivered.

As intensive legume production is currently limited in Europe, advocates for sustainable intensification with legumes look to examples from other parts of the world. Canada’s experience provides a proof of concept (Watson et al., 2017, Magrini et al., 2018). Through state-sponsored breeding programmes in the 1970s and 80 s, the country developed high yielding and climatically suited cultivars. Owing to up-take by large-scale and hi-tech agricultural operations, the country is now the leading exporter of many different legumes – primarily high-quality produce marketed for human consumption (Zentner et al., 2002). Importantly, legumes are an element of crop rotations, and their rapid rise has not been geared towards an environmentally costly, input dependent monoculture. For example, in the Saskatchewan region, one of Canada’s major arable area, lentils account for 10% of the cropped area and other grain legumes for another 10% (Watson et al., 2017). In this region, the increased representation of legumes has improved farm profitability and environmental performance (Luce et al., 2015). Other success stories of the sustainable intensification of legumes from Turkey and Pakistan (Byerlee and White, 2000) informed a roadmap for how Europe could benefit from increasing the representation of legumes in its arable agriculture (Lesschen et al., 2011, Watson et al., 2017).

Advocates for agroecology in Europe have a very different vision for legumes (Badgley and Perfecto, 2007, Gogoi et al., 2018). In this food future, they play key role in the development of low-impact, organic or veganic (stockless organic systems) to meet the considerable task of producing enough food for a large and growing population. For example, Jensen et al. (2007) report on the positive outcomes of an EU funded research project looking into the capacity for organic legume-cereal intercropping and rotations to increase yield, decrease nitrogen losses, and improve GHG emissions relative to mineral fertiliser-based systems. Similarly, Lampkin et al. (2015) review how agroecological methods – including the use of legumes in rotations in conjunction with no-till management or intercropping – can help improve the environmental performance of European farming whilst not jeopardising its food security. In these agroecological accounts, legumes find good fit with the eco-pessimist enthusiasm for the revitalisation of local and traditional management practices. They draw attention to how before the advent of mineral fertilisers, European farming relied on fertilisation from legume crops and organic manure (Magrini et al., 2016). They suggest that increasing the role of legumes will not only advance sustainability but also enable a return to a desired pre-industrialised model of European agriculture, helping to resist the logics of ongoing agricultural intensification. Articulations of the enmeshment of legumes’ political and agronomic potential from ‘alternative’ farming voices can be seen at Landworkers’ Alliance (2019) and La Via Campesina (2017).

In contrast to sustainable intensification, the flows of knowledge about best practice and management innovation in agroecology flow from the South to the North. Knowledge and experience of agroecological practices, profitability, and environmental performance originating in smallholdings across sub-Saharan Africa (Belmain et al., 2013), Central America (Bunch 1999; Alteiri and Nicholls, 2008), and South-East Asia (Monyo and Gowda, 2014) form the mainstay of the current research base. Advocates highlight how these findings demonstrate how legumes displace the need to buy into international supply chains of mineral fertiliser provision to achieve the wider ideals of agroecology for local food production and distribution, food sovereignty, and food security (Altieri, 2009)*.* In this regard, advocates suggest that legumes can help increase European protein self-sufficiency in providing feed for the livestock sector (Watson et al., 2017) and plant-based proteins for human markets (Murphy-Bokern et al., 2017, European Commission 2018) – addressing two major challenges in the European food system.

Tracing the promises of legumes makes clear the intersectionality between the visions for sustainable intensification and agroecology. Both paradigms are looking to decouple production from environmental damage, using legumes to displace the need for mineral fertilisation either as part of a large-scale, hi-tech, consolidated and international food system, or as part of robust local, low-impact systems whose products are distributed through alternative food networks. The overlap between the ideas of agroecology and SI (and related terms such as regenerative and conservation agriculture) has been noted elsewhere (Wezel et al., 2015). Their shared objectives facilitate a porosity in determining which management practices pertain to what paradigm (Tittonell 2014) and legumes become a point of consensus amongst authors looking to stress how agroecological practices can help meet sustainable intensification objectives, and how sustainable intensification principles can be used to scale-up agroecological systems (Lampkin et al., 2015, Altieri et al., 2017).

## 6 Conclusions: What futures for legumes in Europe?

In mapping legumes’ promissory narratives, we have revealed the diverse ‘agrarian dreams’ (Guthman 2014) that they permit. This diversity can be read as a promiscuity: the varied health and environmental applications of legumes in food processing, farming, and diets mean that different value judgments on what constitutes a ‘good’ food system can be advanced by calls for their greater representation. In these diffracted, kaleidoscopic perspectives, legumes are grown on small biodiverse farms reminiscent of low-intensity, pre-industrial European agriculture. They fill the fields of large-scale intensive plantations, linked by globalised agricultural systems. Legumes are the future of green livestock, and they also herald its demise. They are eaten whole, driving a culinary revival of local produce and home cooking, while in other visions they bulk out processed foods, and secure the future of diets based on healthy, high-protein, fast or convenient foods. In all of these food futures, legumes help save the environment; but this analysis makes clear that there are many, very different environments that they are working to save. If, as advocates signal, legumes are enjoying a ‘coming of age’ moment (Considine et al., 2017), then this paper has established an important imperative for legume researchers to recognise the contrasting and sometimes conflicting desires to which their dreams give expression. Food futurology is addictive and can be narcissistic, and we don’t want anyone going mad from frustrated wish fulfilment.

Rather than conclude this paper with cod psychoanalysis, we will briefly draw out three key areas of consensus that cut across the six promissory narratives we have sketched above. First, all advocates agree that the potential of legumes to fix nitrogen can help both cropping and grass systems move away from the current reliance on mineral fertilisation (Lampkin et al., 2015, Reckling et al., 2016). They can reduce the GHG emissions associated with fertiliser production and application, as well as secure more local environmental improvements such as water quality and biodiversity (Nemecek et al., 2008, Lötjönen and Ollikainen, 2017). Second, proponents agree that legumes have desirable nutritional qualities: they are high in protein and contain particular amino acids (notably lysine) otherwise difficult to source in plant-based diets. Legumes have the potential to play a central role in healthy and sustainable human diets (Davis et al., 2010, Springmann et al., 2018) and to enhance the quality of animal feed (Watson et al., 2017). All agree that this quality is economically significant at a time when consumers, processors, retailers and food influencers are increasingly beguiled by protein as a dietary component (Katz et al., 2019).

Finally advocates agree that the historicity of legumes – in terms of their long-standing roles in the European food system as a human foodstuff, as animal feed, and as a source of fertilisation – helps softens their image, making them socially acceptable for future models of sustainable food and farming. Legumes were often grown within the living memory of European farmers, and scattered collective knowledge remains on how legumes can be enrolled in different management systems. Farming is risk-averse and conservative and European farmers place considerable stock in generational and localised knowledge systems (Cush and Macken-Walsh, 2016, Wójcik et al., 2019). Advocates agree that legumes might be promoted without attracting the resistance associated with more novel sustainable food-production practices (Šūmane et al., 2018). From the consumption side – and leaving aside the opinions of some techno-optimists – most agree that consumers will remain more willing to embrace plant-based products over novel insect or cellular meat alternatives (Siegrist et al., 2018, Gómez-Luciano et al., 2019). Here legumes’ stigma as ‘poor man’s meat’ works in their favour. Food marketers working on plant-based meat-analogues, whole and unprocessed pulses, or biofortified foods can promote legumes as unremarkable and familiar products whose wholesome provenance serves to reassure the majority of cautious and conservative consumers.

While this paper has focused on the ideological differences between contrasting legume dreams, there are important points of consensus. We wager that the map of promissory narratives offered by this paper will help deliberations amongst researchers, policymakers, farmers and businesses engaged in making the futures of plant-based food in Europe.

## CRediT authorship contribution statement

**George Cusworth:** Conceptualisation, Methodology, Software, Investigation, Formal analysis, Writing - original draft. **Tara Garnett:** Resources, Validation, Writing - review & editing, Supervision. **Jamie Lorimer:** Conceptualisation, Validation, Resources, Writing - review & editing, Supervision.

## Declaration of Competing Interest

The authors declare that they have no known competing financial interests or personal relationships that could have appeared to influence the work reported in this paper.

## References

[b0005] Allen R.C. (2008). The nitrogen hypothesis and the English agricultural revolution: A biological analysis. J. Econ. Hist..

[b0010] Altieri M.A. (2009). Agroecology, small farms, and food sovereignty. Mon. Rev..

[b0015] Altieri M.A., Nicholls C.I., Montalba R. (2017). Technological approaches to sustainable agriculture at a crossroads: an agroecological perspective. Sustainability.

[b0020] Ambler-Edwards, S., Bailey, K., Kiff, A., Lang, T., Lee, R., Marsden, T., Simons, D., Tibbs, H., (2009), Food futures: rethinking UK strategy, A report by Chatham House.

[b0025] Anderson J.W., Major A.W. (2002). Pulses and lipaemia, short-and long-term effect: potential in the prevention of cardiovascular disease. Br. J. Nutr..

[b0030] Asif M., Rooney L.W., Ali R., Riaz M.N. (2013). Application and opportunities of pulses in food system: a review. Crit. Rev. Food Sci. Nutr..

[b0035] Badgley C., Perfecto I. (2007). Can organic agriculture feed the world?. Renewable Agric. Food Syst..

[b0040] Basu, P., Scholten, B.A. (2012). Technological and social dimensions of the Green Revolution: connecting pasts and futures.

[b0045] Becerra-Tomás N., Babio N., Martínez-González M.Á., Corella D., Estruch R., Ros E., Bellarby J., Tirado R., Leip A., Weiss F., Lesschen J.P., Smith P. (2013). Livestock greenhouse gas emissions and mitigation potential in Europe. Global Change Biology.

[b0050] Bellarby J., Tirado R., Leip A., Weiss F., Lesschen J.P., Smith P. (2013). Livestock greenhouse gas emissions and mitigation potential in Europe. Glob. Change Biol..

[b0055] Belmain, S. R., Haggar, J., Holt, J., & Stevenson, P. C. (2013). Managing legume pests in sub-Saharan Africa: Challenges and prospects for improving food security and nutrition through agro-ecological intensification.

[b9010] Bentrup, F., Palliere, C., 2008, GHG Emissions and Energy Efficiency in European Nitrogen Fertiliser Production and Use, The International Fertiliser Society, Proceedings Number 639.

[b0060] Bernard B., Lux A. (2017). How to feed the world sustainably: an overview of the discourse on agroecology and sustainable intensification. Reg. Environ. Change.

[b0065] Beverland M.B. (2014). Sustainable eating: mainstreaming plant-based diets in developed economies. Journal of Macromarketing.

[b0070] Boland M.J., Rae A.N., Vereijken J.M., Meuwissen M.P., Fischer A.R., van Boekel M.A., Hendriks W.H. (2013). The future supply of animal-derived protein for human consumption. Trends Food Sci. Technol..

[b0075] Birt L., Scott S., Cavers D., Campbell C., Walter F. (2016). Member checking: a tool to enhance trustworthiness of merely a not to validation?. Qual. Health Res..

[b0080] Boye J., Zare F., Pletch A. (2010). Pulse proteins: Processing, characterization, functional properties and applications in food and feed. Food Res. Int..

[b0085] Budgar L. (2017). Veganism on the Rise. *Amass*.

[b0090] Brown N., Michael M. (2003). A sociology of expectations: retrospecting prospects and prospecting retrospects. Technol. Anal. Strategic Manage..

[b0095] Bunch R. (1999). More productivity with fewer external inputs: Central American case studies of agroecological development and their broader implications. Environ. Dev. Sustain..

[b0100] Byerlee D., White R. (2000). Agricultural systems intensification and diversification through food legumes: technological and policy options.

[b0105] Clay N., Sexton A.E., Garnett T., Lorimer J. (2020). Palatable disruption: the politics of plant milk. Agric. Hum. Values.

[b9005] Considine M.J., Siddique K.H.M., Foyer C.H. (2017). Nature's pulse power: legumes, food security and climate change. J. Exp. Bot..

[b0110] Curran J. (2012). The nutritional value and health benefits of pulses in relation to obesity, diabetes, heart disease and cancer. Br. J. Nutr..

[b0115] Cush P., Macken-Walsh Á. (2016). Farming ‘through the ages’: joint farming ventures in Ireland. Rural Society.

[b0120] Daemmgen U., Schulz J., Klausing H.K., Hutchings N.J., Haenel H.D., Roesemann C. (2012). Enteric methane emissions from German pigs. Landbauforschung Völkenrode.

[b0125] Dagevos H. (2014). Flexibility in the frequency of meat consumption–empirical evidence from the Netherlands. EuroChoices.

[b0130] Katz D., Doughty K., Geagan K., Jenkins D., Gardner C. (2019). Perspective: The Public Health Case for Modernizing the Definition of Protein Quality. Advances in Nutrition.

[b0135] Davis J., Sonesson U., Baumgartner D.U., Nemecek T. (2010). Environmental impact of four meals with different protein sources: case studies in Spain and Sweden. Food Res. Int..

[b0140] Day L. (2013). Proteins from land plants–potential resources for human nutrition and food security. Trends Food Sci. Technol..

[b0145] de Boer J., Helms M., Aiking H. (2006). Protein consumption and sustainability: diet diversity in EU-15. Ecol. Econ..

[b0150] de Souza Monteiro, D., Brandt, K., Cooper, J., Panzone, L., Watson, A., Cheng, Y., Benedetti, E., (2017). Opportunities and challenges for the development of pulses markets, Report Comission by PGRO and N8 Innovation Forum.

[b0155] Dicks L., Rose D., Ang F. (2018). What agricultural practices are most likely to deliver “sustainable intensification” in the UK?. Food Energy Secur..

[b0160] Dubuisson-Quellier S., Lamine C. (2008). Consumer involvement in fair trade and local food systems: delegation and empowerment regimes. GeoJournal.

[b0165] Ebert A.W. (2014). Potential of underutilized traditional vegetables and legume crops to contribute to food and nutritional security, income and more sustainable production systems. Sustainability.

[b0170] Ellis J.L., Bannink A., France J., Kebreab E., Dijkstra J. (2010). Evaluation of enteric methane prediction equations for dairy cows used in whole farm models. Glob. Change Biol..

[b0175] European Commission (2018), Report from The Commission to the council and the European Parliament on the Development of Plant Proteins in the EU, European Commission COM(2018)-757.

[b0180] Euvepro (2019), THE USE OF PLANT-BASED PROTEINS IN FOOD AND BEVERAGES IN THE EU A 10-year review of New Product Launches Containing Plant-Based Proteins across EU 28, accessed via the Euvropro website, June 2020.

[b0185] Evans A., Miele M. (2012). Between food and flesh: How animals are made to matter (and not matter) within food consumption practices. Environ. Plann. D: Soc. Space.

[b0190] Farmers Guardian What is the most sustainable way to produce meat and milk? Published online at 2020 https://www.fginsight.com/news/news/what-is-the-most-sustainable-way-to-produce-meat-and-milk-106540, accessed February 2021.

[b0195] Flint-Hamilton K.B. (1999). Legumes in ancient Greece and Rome: food, medicine, or poison?. Hesperia: The Journal of the American School of Classical Studies at Athens.

[b0200] Food and Agriculture Organization of the United Nations (FAO) (2016). Pulses: Nutritious Seeds for a Sustainable Future, Published by the FAO, ISBN: 978-92-5-109172-2.

[b0205] Foyer C.H., Lam H.M., Nguyen H.T., Siddique K.H., Varshney R.K., Colmer T.D., Cooper J.W. (2016). Neglecting legumes has compromised human health and sustainable food production. Nat. Plants.

[b0210] Fraanje W., Garnett T. (2020).

[b0215] Friedrich T. (2015). A new paradigm for feeding the world in 2050 the sustainable intensification of crop production. Resource Magazine.

[b0220] Friends of the Earth, (2012), A wolf in sheep’s clothing? Published online at https://www.foei.org/wp-content/uploads/2013/12/Wolf-in-Sheeps-Clothing-for-web.pdf, accessed February 2021.

[b0225] FWI – Farmers Weekly, key findings of EU sustainable intensification report, published online at 10 2014 https://www.fwi.co.uk/news/environment/10-key-findings-of-eu-sustainable-intensification-report, accessed February 2021.

[b0230] FWI – Farmers Weekly (2020), Advice on establishing and grazing herbal lays, published online at https://www.fwi.co.uk/livestock/grassland-management/advice-on-establishing-and-grazing-herbal-leys, accessed February 2021.

[b0235] Gadanakis Y., Bennett R., Park J., Areal F.J. (2015). Improving productivity and water use efficiency: A case study of farms in England. Agric. Water Manag..

[b0240] Galloway J.N., Townsend A.R., Erisman J.W., Bekunda M., Cai Z., Freney J.R., Sutton M.A. (2008). Transformation of the nitrogen cycle: recent trends, questions, and potential solutions. Science.

[b0245] Gambert, I. (2019). Got Mylk? The Disruptive Possibilities of Plant Milk. The Disruptive Possibilities of Plant Milk (May 15, 2019), 84.

[b0250] Gan Y., Hamel C., O’Donovan J.T., Cutforth H., Zentner R.P., Campbell C.A., Poppy L. (2015). Diversifying crop rotations with pulses enhances system productivity. Sci. Rep..

[b0255] Garnett T. (2009). Livestock-related greenhouse gas emissions: impacts and options for policy makers. Environ. Sci. Policy.

[b0260] Gerbens-Leenes P.W., Nonhebel S. (2002). Consumption patterns and their effects on land required for food. Ecol. Econ..

[b0265] Gerber P.J., Steinfeld H., Henderson B., Mottet A., Opio C., Dijkman J., Falcucci A., Tempio G. (2013). Food and Agriculture Organization of the United Nations (FAO).

[b0270] Gibson-Graham J.K. (2006).

[b0275] Gliessman S.R. (2007).

[b0280] Global Food Security, (2017), The carbon footprint of high-protein foods, Policy Lap Report by GFS.

[b0285] Godfray H.C.J., Beddington J.R., Crute I.R., Haddad L., Lawrence D., Muir J.F., Pretty J., Robinson S., Thomas S., Toulmin C. (2010). Food security: the challenge of feeding 9 billion people. Science.

[b0290] Godfray H.C.J., Garnett T. (2014). Food security and sustainable intensification. Philos. Trans. Royal Soc. B: Biol. Sci..

[b0295] Gogoi N., Baruah K.K., Meena R.S. (2018). Legumes for Soil Health and Sustainable Management.

[b0300] Goldstein J. (2018).

[b0305] Gómez-Luciano C.A., de Aguiar L.K., Vriesekoop F., Urbano B. (2019). Consumers’ willingness to purchase three alternatives to meat proteins in the United Kingdom, Spain, Brazil and the Dominican Republic. Food Qual. Prefer..

[b0310] Goodman M.K. (2004). Reading fair trade: Political ecological imaginary and the moral economy of fair trade foods. Political Geography.

[b0315] Gruber N., Galloway J.N. (2008). An Earth-system perspective of the global nitrogen cycle. Nature.

[b0320] Guardian The (2019). Can we ditch intensive farming – and still feed the world?. Published online at.

[b0325] Guthman J. (2014).

[b0330] Hamann, K., Tran, F., Iannetta, P., Duarte, M., Pinto, E., Vasconcelos, M., Williams, M., Szuromi O., Kraul, A., Gabor, B., Lehrach, U., (2019) Business cases, published through the TRUE Research Project website, https://www.true-project.eu/publications-resources/publications/, accessd February 2021.

[b0335] Harper, K., Alfonso, A., (2019). Food values in a Lisbon Urban Garden: Between Sabor, Saber and the Market, in Food Values in Europe, Siniscalchi, V., Harper, K., (Eds.) Bloomsbury Academic Publishing.

[b0340] Hedley C.L. (2001). Carbohydrates in grain legume seeds. Improving Nutritional Quality and Agronomic Characteristics.

[b0345] Herrero M., Gerber P., Vellinga T., Garnett T., Leip A., Opio C., Montgomery H. (2011). Livestock and greenhouse gas emissions: The importance of getting the numbers right. Anim. Feed Sci. Technol..

[b0350] Hexa (2019) Global Legumes Market Size And Forecast, By Type (Beans, Peas, Nuts, Others) By Region (North America, Europe, Asia Pacific, Central and South America and Middle East and Africa) And Trend Analysis, 2019 – 2025, Accessed June 2020, https://www.hexaresearch.com/research-report/legumes-market.

[b0355] Holt-Giménez E., Altieri M.A. (2013). Agroecology, food sovereignty, and the new green revolution. Agroecology and Sustainable Food Systems.

[b0360] Horlings L.G., Marsden T.K. (2011). Towards the real green revolution? Exploring the conceptual dimensions of a new ecological modernisation of agriculture that could ‘feed the world’. Global Environ. Change.

[b0365] Hörtenhuber S.J., Lindenthal T., Zollitsch W. (2011). Reduction of greenhouse gas emissions from feed supply chains by utilizing regionally produced protein sources: the case of Austrian dairy production. J. Sci. Food Agric..

[b0370] Ionel I. (2017). The Effect of Coupled Subsidies on the Romanian Soybean Market. Agricultural Economics and Rural Development.

[b0375] Iqbal A., Khalil I.A., Ateeq N., Khan M.S. (2006). Nutritional quality of important food legumes. Food Chem..

[b0380] Jasanoff S., Jasanoff S., Kim S.-.H. (2015). Dreamscapes of Modernity.

[b0385] Jensen E.S., Ambus P., Bellostas N., Boisen S., Brisson N., Corre-Hellou G., Gooding M. (2007). In *1st European Joint Organic Congress*.

[b0390] Jha A.B., Warkentin T.D. (2020). Biofortification of pulse crops: Status and future perspectives. Plants.

[b0395] Jones A.D., Ejeta G. (2016). A new global agenda for nutrition and health: the importance of agriculture and food systems. Bull. World Health Organ..

[b0400] Joshi P., Rao P. (2017). Global pulses scenario: Status and outlook. Ann. N. Y. Acad. Sci..

[b0405] Joshi V.K., Kumar S. (2015). Meat Analogues: Plant based alternatives to meat products-A review. Int. J. Food Ferment. Technol..

[b0410] Kawa, N., (2020), A mend to the metabolic rift? The promises (and potential pitfalls) of biosolids application on American soils, Chapter 9 in Salazar, J., Granjou, C., Kearnes, (Eds.), Thinking with soils, Bloomsbury Collections.

[b0415] Kearnes, M., Rickards, L., (2020), Knowing earth, knowing soil: epistemological work and the political aesthetics of regenerative agriculture, Chapter 5 in Salazar, J., Granjou, C., Kearnes, (Eds.), Thinking with soils, Bloomsbury Collections.

[b0420] Kitson, A., Marshall, A., Basssett, K., Zeitz, K., (2012), What are the core elements of patient-centred care? A narrative review and synthesis of the literature from health policy, medicine and nursing, Journal of Advanced Nursing, 69 (1), 4-15. 10.1111/j.1365-2648.2012.06064.x 22709336

[b0425] Kulak M., Nemecek T., Frossard E., Chable V., Gaillard G. (2015). Life cycle assessment of bread from several alternative food networks in Europe. J. Cleaner Prod..

[b0430] Kumar S., Pandey G. (2020). Biofortification of pulses and legumes to enhance nutrition. Heliyon.

[b0435] La Poutré, H.J. (2015). The contribution of legumes to the diet of English peasants and farm servants, c. 1300. Agricultural History Review, 63(1), 19-38.

[b0440] La Via Campesina, (2017), Toolkit: peasant agroecology schools and the peasant-to-peasant method of horizontal learning, published online at https://foodfirst.org/wp-content/uploads/2017/06/TOOLKIT_agroecology_Via-Campesina-1.pdf, accessed February 2021.

[b0445] Landworkers’ Alliance, (2014), Feeding the Future, published at https://landworkersalliance.org.uk/wp-content/uploads/2018/10/Feeding-the-Future-Landworkers-Alliance-A4-low-res.pdf, accessed February 2021.

[b0450] Landworkers’ Alliance (2019), Food, farming and the climate crisis: how we can feed people and cool the planet, published online at https://orfc.org.uk/wp-content/uploads/2020/12/Farming-Food-and-the-Climate-Crisis_v2-Catherine-McAndrew.pdf, accessed February 2021.

[b0455] Lampkin, N., Pearce, B., Leake, A., Creissen, H., Gerrard, C. L., Gerling, R., & Vieweger, A. (2015). The role of agroecology in sustainable intensification.

[b0460] Lavallée M., Robillard P., Mirsalari R. (2013). Performing Systematic Literature Reviews With Novices: An Iterative Approach. IEEE Trans. Educ..

[b0465] Ledgard S., Schils R., Eriksen J., Luo J. (2009). Environmental impacts of grazed clover/grass pastures. Irish J. Agric. Food Res..

[b0470] Leip, A., Weiss, F., Wassenaar, T., Perez, I., Fellmann, T., Loudjani, P.,& Biala, K. (2010). Evaluation of the livestock sector's contribution to the EU greenhouse gas emissions (GGELS).

[b0475] Lesschen J.P., Van den Berg M., Westhoek H.J., Witzke H.P., Oenema O. (2011). Greenhouse gas emission profiles of European livestock sectors. Anim. Feed Sci. Technol..

[b0480] Lockie S. (2009). Responsibility and agency within alternative food networks: assembling the “citizen consumer”. Agric. Hum. Values.

[b0485] Lötjönen S., Ollikainen M. (2017). Does crop rotation with legumes provide an efficient means to reduce nutrient loads and GHG emissions?. *Review of Agricultural, Food and Environmental Studies*.

[b0490] Luce M.S., Grant C.A., Zebarth B.J., Ziadi N., O’Donovan J.T., Blackshaw R.E., May W.E. (2015). Legumes can reduce economic optimum nitrogen rates and increase yields in a wheat–canola cropping sequence in western Canada. Field Crops Research.

[b0495] Lupton D. (2017). ‘Download to delicious’: Promissory themes and sociotechnical imaginaries in coverage of 3D printed food in online news sources. Futures.

[b0500] Lüscher A., Mueller-Harvey I., Soussana J.F., Rees R.M., Peyraud J.L. (2014). Potential of legume-based grassland–livestock systems in Europe: a review. Grass Forage Sci..

[b0505] Lynas M. (2011). The God species: how the planet can survive the age of humans. Fourth.

[b0510] Macdiarmid J.I., Douglas F., Campbell J. (2016). Eating like there's no tomorrow: Public awareness of the environmental impact of food and reluctance to eat less meat as part of a sustainable diet. Appetite.

[b0515] Magrini M.B., Anton M., Chardigny J.M., Duc G., Duru M., Jeuffroy M.H., Walrand S. (2018). Pulses for sustainability: breaking agriculture and food sectors out of lock-in. Frontiers in Sustainable Food Systems.

[b0520] Magrini M.B., Anton M., Cholez C., Corre-Hellou G., Duc G., Jeuffroy M.H., Walrand S. (2016). Why are grain-legumes rarely present in cropping systems despite their environmental and nutritional benefits? Analyzing lock-in in the French agrifood system. Ecol. Econ..

[b0525] Mahmood, F., Shahzad, T., Hussain, S., Shahid, M., Azeem, M., & Wery, J. (2018). Grain legumes for the sustainability of european farming systems. In Sustainable Agriculture Reviews 32 (pp. 105-133). Springer, Cham.

[b0530] Mahon N., Crute I., Simmons E., Islam M.M. (2017). Sustainable intensification–“oxymoron” or “third-way”? A systematic review. Ecol. Ind..

[b0535] Makri E., Papalamprou E., Doxastakis G. (2005). Study of functional properties of seed storage proteins from indigenous European legume crops (lupin, pea, broad bean) in admixture with polysaccharides. Food Hydrocolloids.

[b0540] Mann S., Necula R. (2020). Are vegetarianism and veganism just half the story? Empirical insights from Switzerland. British Food Journal..

[b0545] Margier M., Georgé S., Hafnaoui N., Remond D., Nowicki M., Du Chaffaut L., Reboul E. (2018). Nutritional composition and bioactive content of legumes: Characterization of pulses frequently consumed in France and effect of the cooking method. Nutrients.

[b0550] McCrory M.A., Hamaker B.R., Lovejoy J.C., Eichelsdoerfer P.E. (2010). Pulse consumption, satiety, and weight management. Advances in Nutrition.

[b0555] McDermott J., Wyatt A.J. (2017). The role of pulses in sustainable and healthy food systems. Ann. N. Y. Acad. Sci..

[b0560] Méndez V.E., Bacon C.M., Cohen R. (2013). Agroecology as a transdisciplinary, participatory, and action-oriented approach. Agroecology and Sustainable Food Systems.

[b0565] Metzger, M.J., Murray-Rust, D., Houtkamp, J., Jensen, A., La Riviere, I., Paterson, J. S., & Valluri-Meynard, J. M. (2013). Innovating in cropping and farming systems, in Coudel, E., Devautour, H., Soulard, C., Faure, G., Hubert, B., (Eds.), Renewing innovation systems in agriculture and food, Wageningen Academic Publishers, Wageningen.

[b0575] Meynard J.-M., Charrier F., Fares M., Le Bail M., Magrini M.-B., Charlie A., Messean A. (2018). Socio-technical lock-in hinders crop diversification in France. Agron. Sustainable Dev..

[b0580] Monyo E.S., Gowda C.L. (2014).

[b0585] Morris C. (2018). ‘Taking the politics out of broccoli’: debating (de) meatification in UK national and regional newspaper coverage of the Meat Free Mondays campaign. Sociologia Ruralis.

[b0590] Mudryj A.N., Yu N., Aukema H.M. (2014). Nutritional and health benefits of pulses. Appl. Physiol. Nutr. Metab..

[b0595] Murphy-Bokern, D. O. N. A. L., Peeters, A. L. A. I. N., & Westhoek, H. E. N. K. (2017). The role of legumes in bringing protein to the table. D, M.-B., FL, S. & C, W.(eds) Legumes in Cropping Systems CABI Publishing, 18-36.

[b0600] Nemecek T., von Richthofen J.S., Dubois G., Casta P., Charles R., Pahl H. (2008). Environmental impacts of introducing grain legumes into European crop rotations. Eur. J. Agron..

[b0605] Neuenfeldt S., Gocht A., Heckelei T., Ciaian P. (2019). Explaining farm structural change in the European agriculture: a novel analytical framework. European Review of Agricultural Economics.

[b0610] Newman, L. (2020). The Promise and Peril of “Cultured Meat”. Green Meat?: Sustaining Eaters Animals and the Planet, 169.

[b0615] NFU (National Farmers’ Union) (2016), Sustainable intensification update, published online at https://www.nfuonline.com/cross-sector/science-and-technology/research-and-innovation-news/sustainable-intensification-update/, accessed February 2021.

[b0620] Oliveira, B., de Moura, A. P., & Cunha, L. M. (2019). Increasing Pulse Consumption to Improve Human Health and Food Security and to Mitigate Climate Change. In Castro, P., Azul, A., Leal Filho, W., Azeiteiro, U. (Eds.), Climate Change-Resilient Agriculture and Agroforestry. Springer, International Publishing.

[b0625] Oliveira G., Hecht S. (2016). Sacred groves, sacrifice zones and soy production: globalization, intensification and neo-nature in South America. Journal of Peasant Studies.

[b0630] ORC – Organic Research Centre (2018), Livestock on diverse leys: a return to the past for a promising future, published online at https://www.agricology.co.uk/sites/default/files/ORC126_Mullender_1.pdf, accessed February 2021.

[b0635] Orlando, G. (2018). Understanding Alternative Food Networks After the Crisis: Testing Four Scenarios in Italy, pp. pp. 137-162 in Corsi, A., Barbera, F., Dansero, E., Peano, C., (Eds.), Alternative Food Networks. Palgrave Macmillan.

[b0640] Parham J. (2015).

[b0645] Pellett P.L., Ghosh S. (2004). Lysine fortification: past, present, and future. Food Nutr. Bull..

[b0650] Pohjolainen P., Jokinen P. (2020). Meat reduction practices in the context of a social media grassroots experiment campaign. Sustainability.

[b0655] Preissel S., Reckling M., Schläfke N., Zander P. (2015). Magnitude and farm-economic value of grain legume pre-crop benefits in Europe: a review. Field Crops Research.

[b0660] Pretty, J.N. (1997). The sustainable intensification of agriculture. In Natural resources forum (Vol. 21, No. 4, pp. 247-256). Oxford, UK: Blackwell Publishing Ltd.

[b0665] Pretty J., Benton T.G., Bharucha Z.P., Dicks L.V., Flora C.B., Godfray H.C.J., Pierzynski G. (2018). Global assessment of agricultural system redesign for sustainable intensification. Nat. Sustainability.

[b0670] Pulses U.K. (2020). Eat more British beans!. Accessed.

[b0675] Radkau J. (2014).

[b0680] Rebanks J. (2020).

[b9020] Rebello C.J., Greenway F.L., Finley J.W. (2014). Nutrition and health benefits of legumes. Obesity Rev..

[b0685] Reckling M., Bergkvist G., Watson C.A., Stoddard F.L., Zander P.M., Walker R.L., Bachinger J. (2016). Trade-offs between economic and environmental impacts of introducing legumes into cropping systems. Front. Plant Sci..

[b0690] Redman, G., (2015), Revealing the opportunities for growing peas and beans in the UK, a report by The Andersons Centre for the John Innes Centre, available through www.theandersonscentre.co.uk website, accessed June 2020.

[b0695] Research and Markets (2020). Global pulse ingredients market size, market share, application analysis, regional outlook, growth trends, key players, competitive strategies and forecasts, 2019 to 2027. http://2020.

[b0700] Rizkalla S.W., Bellisle F., Slama G. (2002). Health benefits of low glycaemic index foods, such as pulses, in diabetic patients and healthy individuals. Br. J. Nutr..

[b0705] Robinson R.A., Sutherland W.J. (2002). Post-war changes in arable farming and biodiversity in Great Britain. J. Appl. Ecol..

[b0710] Rochon J.J., Doyle C.J., Greef J.M., Hopkins A., Molle G., Sitzia M., Smith C.J. (2004). Grazing legumes in Europe: a review of their status, management, benefits, research needs and future prospects. Grass Forage Sci..

[b0715] Röös E., Bajželj B., Smith P., Patel M., Little D., Garnett T. (2017). Greedy or needy? Land use and climate impacts of food in 2050 under different livestock futures. Global Environ. Change.

[b0720] Röös E., Carlsson G., Ferawati F., Hefni M., Stephan A., Tidåker P., Witthöft C. (2018). Less meat, more legumes: prospects and challenges in the transition toward sustainable diets in Sweden. Renewable Agric. Food Syst..

[b0725] Rosenfeld D.L., Burrow A.L. (2017). Vegetarian on purpose: Understanding the motivations of plant-based dieters. Appetite.

[b0730] Sasu-Boakye Y., Cederberg C., Wirsenius S. (2014). Localising livestock protein feed production and the impact on land use and greenhouse gas emissions. Animal.

[b0735] Sexton A.E., Garnett T., Lorimer J. (2019). Framing the future of food: The contested promises of alternative proteins. Environment and Planning E: Nature and Space.

[b0740] Seyfang G. (2006). Ecological citizenship and sustainable consumption: Examining local organic food networks. J. Rural Stud..

[b0745] Siegrist M., Sütterlin B., Hartmann C. (2018). Perceived naturalness and evoked disgust influence acceptance of cultured meat. Meat Sci..

[b0750] Smart A. (2004). Adrift in the mainstream: Challenges facing the UK vegetarian movement. British Food Journal..

[b0755] Springmann M., Wiebe K., Mason-D'Croz D., Sulser T.B., Rayner M., Scarborough P. (2018). Health and nutritional aspects of sustainable diet strategies and their association with environmental impacts: a global modelling analysis with country-level detail. The Lancet Planetary Health.

[b0760] Squire G., Quesada N., Begg G., Iannetta P. (2019). Transitions to legume inclusion in cropland: defining opportunities and estimating benefits for the nitrogen economy, *Food and Energy*. Security.

[b0765] Stagnari F., Maggio A., Galieni A., Pisante M. (2017). Multiple benefits of legumes for agriculture sustainability: an overview. Chemical and Biological Technologies in Agriculture.

[b0770] Staniszewski J. (2018). Attempting to measure sustainable intensification of agriculture in countries of the European Union. J. Environ. Prot. Ecol..

[b0775] Steinfeld H., Gerber P. (2010). Livestock production and the global environment: Consume less or produce better?. Proc. Natl. Acad. Sci..

[b0780] Stephens, N.S. (2015). What is in vitro meat? Food phreaking.

[b0785] Stinner D.H., Glick I., Stinner B.R. (1992). Forage legumes and cultural sustainability: lessons from history. Agric. Ecosyst. Environ..

[b0790] Šūmane S., Kunda I., Knickel K., Strauss A., Tisenkopfs T., des Ios Rios, I., & Ashkenazy, A (2018). Local and farmers' knowledge matters! How integrating informal and formal knowledge enhances sustainable and resilient agriculture. Journal of Rural Studies.

[b0795] Tharanathan R.N., Mahadevamma S. (2003). Grain legumes—a boon to human nutrition. Trends Food Sci. Technol..

[b0800] Tittonell P. (2014). Ecological intensification of agriculture—sustainable by nature. Current Opinion in Environmental Sustainability.

[b0805] Tomlinson I. (2013). Doubling food production to feed the 9 billion: a critical perspective on a key discourse of food security in the UK. Journal of Rural Studies.

[b0810] Trinidad T.P., Mallillin A.C., Loyola A.S., Sagum R.S., Encabo R.R. (2010). The potential health benefits of legumes as a good source of dietary fibre. Br. J. Nutr..

[b0815] Tudisca S., Di Trapani A.M., Sgroi F., Testa R., Giamporcaro G. (2014). Role of alternative food networks in Sicilian farms. Int. J. Entrepreneurship Small Bus..

[b0820] Voisin A.S., Guéguen J., Huyghe C., Jeuffroy M.H., Magrini M.B., Meynard J.M., Pelzer E. (2014). Legumes for feed, food, biomaterials and bioenergy in Europe: a review. Agron. Sustainable Dev..

[b0825] Vollman J. (2016). Soybean versus other food grain legumes: A critical appraisal of the United Nations Year of the Pulses 2016. Die Bodenkultur: Journal of Land Management Food and Environment.

[b0830] Vranken L., Avermaete T., Petalios D., Mathijs E. (2014). Curbing global meat consumption: Emerging evidence of a second nutrition transition. Environ. Sci. Policy.

[b0835] Wang S.Y., Huang D.J. (2005). Assessment of greenhouse gas emissions from poultry enteric fermentation. Asian-australasian journal of animal sciences.

[b0840] Watson C.A., Reckling M., Preissel S., Bachinger J., Bergkvist G., Kuhlman T., Zander P. (2017). Grain legume production and use in European agricultural systems. Adv. Agron..

[b0845] Weightman R.M., Cottrill B.R., Wiltshire J.J.J., Kindred D.R., Sylvester-Bradley R. (2011). Opportunities for avoidance of land-use change through substitution of soya bean meal and cereals in European livestock diets with bioethanol coproducts. GCB Bioenergy.

[b9015] Weis T. (2013).

[b0850] Weiss F., Leip A. (2012). Greenhouse gas emissions from the EU livestock sector: a life cycle assessment carried out with the CAPRI model. Agric. Ecosyst. Environ..

[b0855] Weltin M., Zasada I., Piorr A., Debolini M., Geniaux G., Perez O.M., Schulp C.J. (2018). Conceptualising fields of action for sustainable intensification–A systematic literature review and application to regional case studies. Agric. Ecosyst. Environ..

[b0860] Wezel A., Bellon S., Doré T., Francis C., Vallod D., David C. (2009). Agroecology as a science, a movement and a practicev. A review. Agronomy for Sustainable Development.

[b0865] Wezel A., Soboksa G., McClelland S., Delespesse F., Boissau A. (2015). The blurred boundaries of ecological, sustainable, and agroecological intensification: a review. Agron. Sustainable Dev..

[b0870] Wójcik M., Jeziorska-Biel P., Czapiewski K. (2019). Between words: A generational discussion about farming knowledge sources. Journal of Rural Studies.

[b0875] Worster D. (1994).

[b9000] Wu G.-L., Liu Y., Tian F.-P., Shi Z.-H. (2017). Legumes functional group promotes soil organic carbon and nitrogen storage by increasing plant diversity. Land Degrad. Dev..

[b0880] Zander P., Amjath-Babu T.S., Preissel S., Reckling M., Bues A., Schläfke N., Murphy-Bokern D. (2016). Grain legume decline and potential recovery in European agriculture: a review. Agron. Sustainable Dev..

[b0885] Zentner R.P., Wall D.D., Nagy C.N., Smith E.G., Young D.L., Miller P.R., Johnston A.M. (2002). Economics of crop diversification and soil tillage opportunities in the Canadian prairies. Agron. J..

